# Air Quality and Hospital Outcomes in Emergency Medical Admissions with Respiratory Disease

**DOI:** 10.3390/toxics4030015

**Published:** 2016-08-05

**Authors:** Seán Cournane, Richard Conway, Declan Byrne, Deirdre O’Riordan, Bernard Silke

**Affiliations:** 1 Medical Physics and Bioengineering Department, St James’s Hospital, Dublin 8, Ireland; sean.cournane@gmail.com; 2 Department of Internal Medicine, St James’s Hospital, Dublin 8, Ireland; drrichardconway@gmail.com (R.C.); declangbyrne@gmail.com (D.B.); doriordan@stjames.ie (D.O.)

**Keywords:** air pollution, particulate matter, sulphur dioxide, emergency medical admission

## Abstract

Background: The impact of very low levels of air pollutants, particulate matter (PM10) and sulfur dioxide (SO_2_) concentrations, on human health is not well characterized. We examined the outcomes (30-day in-hospital mortality) of emergency hospitalizations of respiratory patients and the level of local pollutants on the day of admission. Methods: All emergency admissions (82,421 episodes in 44,660 patients) were recorded over 13 years (2002–2014) and mortality assessed. The median interquartile ranges (IQR) age was 64.5 (43.9, 78.5) years with the proportion of males at 48.5%. Univariate and multivariate logistic regression was used to examine relationships between pollutant concentration (PM10 and SO_2_) and odds ratio (OR) for 30-day in hospital death, after adjustment for acuity. Results: Mortality related to each pollutant variable assessed (as quintiles of increasing atmospheric concentration). For PM10 mortality, the highest two quintiles concentrations were significantly increased (*p* < 0.001) with univariate ORs of 1.30. For SO_2_, the ORs were 1.32, 1.39, and 1.46, for the top three quintiles. There was also a strong relationship between the underlying respiratory function; with forced expiratory volume (FEV_1_) in 1 second (FEV_1_) ≥ 2.0L at the lowest PM10 quintile, mortality was 6.5% (95% CI: 6.1, 6.9) increasing to 9.5% (95% CI: 9.0, 10.0) at the highest PM10 quintile. For patients with FEV_1_ < 2.0L, the mortality at the lowest PM10 quintile was 9.9% (95% CI: 8.8, 10.9) increasing to 14.2% (95% CI: 12.8, 15.6) at the highest quintile. Conclusion: Despite air quality improvement, there was a clear relationship between pollutant concentration and outcomes for respiratory emergency admissions; additionally, the underlying level of pulmonary function was predictive of in-hospital mortality.

## 1. Introduction

In previous studies, predominantly on the general population, a clear association between particulate matter (PM) air pollution and daily mortality has been identified [1,2,3,4]. Respiratory patients when exposed to increased PM concentrations experience a worsening of symptoms and/or higher morbidity as assessed by emergency room visits or hospital admissions [4,5,6,7,8]. Epidemiological evidence indicates an elevated mortality rate among individuals with chronic obstructive pulmonary disease (COPD) following exposure to PM [9,10]. A previous investigation at our institution, over 30 years ago, showed an association between increased mortality and increased urban air pollution [11]. Thereafter, air quality in this area deteriorated further, resulting in the need for legislation in 1990 which led to the banning of marketing, sale, and delivery of bituminous coal. The air pollution control legislation affected a decrease in the average black smoke concentration for Dublin approximating a reduction of 35.6 μg/m^3^ [12]. Death rates were also assessed with adjusted non-trauma death rates evidencing a decrease by 5.7%, with respiratory and cardiovascular deaths decreasing by 15.5% and 10.3%, respectively, providing solid evidence for the governmental intervention [12].

While the benefit of this intervention has been clear, it is suggested in the literature that air quality even when below accepted standards may still contribute to detrimental health effects [2,3,13,14,15]. The effects of PM and sulfur dioxide (SO_2_) have been shown to be independently predictive of all-cause mortality across European cities [2] and in the acute medicine setting [15]. The proposed World Health Organization (WHO) air quality guidelines suggest levels of 50 μg/m^3^ and 20 μg/ m^3^ for 24-h averages of PM10, and SO_2_, respectively [16,17]. It is acknowledged that such guidelines may reduce adverse health effects; however, their implementation cannot completely protect against such health effects. In reference to a previous investigation of air quality among some of the major cities in Europe [6], Dublin’s levels appear lower, with current 24-h averages of PM10 (<40 μg/m^3^) and SO_2_ (<5 μg/m^3^), as comparable to natural background levels of rural Europe [16,17]. Accordingly, in this work we have focused on emergency medical in-patients, investigating how very low levels of PM10 and SO_2_ associated with in-hospital mortality for those acute medical admissions with respiratory disease. Those with respiratory conditions would be considered to be those with increased susceptibility to adverse effects from air pollution. We further considered how underlying pulmonary function of emergency medical inpatients affected mortality.

## 2. Methods

### 2.1. Background

St James’s Hospital is a secondary care center for emergency medical admissions which serves a catchment area of 270,000 adults. All unselected emergency medical admissions between 2002–2014 (13-year period) were recorded into an anonymized database maintained through the Acute Medical Admissions Unit (AMAU), which have been described in detail elsewhere [18,19,20]. The data was collected by the care team in the normal course of patients’ care, with the data anonymized and with individual patients unidentifiable; thus, ethics approval was not needed.

### 2.2. Data Collection

The anonymized patient database assembles core data from each clinical episode obtained from sources such as the patient administration system, the national hospital in-patient enquiry (HIPE), the patient electronic record, the emergency room and other laboratory systems. The International Classification of Diseases, Ninth Revision, Clinical Modification (ICD-9-CM) has been used for both diagnosis and procedure coding from 1990–2005 and ICD-10-CM since then. The data includes demographic characteristics, dates of admission and discharge, principal and secondary diagnoses (ICD-9-CM/ICD-10-CM), in addition to physiological, hematological, and biochemical parameters. HIPE is a national database of coded discharge summaries from acute public hospitals in Ireland, run by the Economic and Social Research Institute [21].

### 2.3. Air Quality

For the current study, PM10 or SO_2_ data from the last 13 years (2002–2014) from three stations within the catchment area (Winetavern and Coleraine Street or Rathmines,) were recorded daily, according to methods detailed elsewhere [22].

### 2.4. Statistics

Descriptive statistics were calculated for demographic data, including means/standard deviations (SD), medians/interquartile ranges (IQR), or percentages. 30-day in-hospital mortality was examined as the primary outcome and compared with categorical variables using chi-square tests. In the cases of multiple comparisons Scheffe’s comparison statistic was used to adjust for multiplicity. Logistic regression analysis was employed to examine significant outcome predictors (*p* < 0.10) of 30-day in hospital mortality. Adjusted odds ratios (OR) and 95% confidence intervals (CI) were calculated for those significant (*p* < 0.10) model predictors. Stepwise logistic regression was used to examine association between mortality and predictor variables such as acute illness severity [23,24,25], co-morbidity [26], and chronic disabling disease [27], sepsis status [28], and deprivation index according to the quintiles of the Small Area Health Research Unit (SAHRU) deprivation number. Stata (Stata Corporation, v.13.1, College Station, TX, USA) software was utilized with the margins command offering an estimate of adjusted predictions for key predictors interactions, while controlling for other variables. Throughout, a *p*-value less than 0.05 was assumed for statistical significance.

## 3. Results

### 3.1. Patient Characteristics

A total of 82,421 episodes, recorded in 44,660 unique patients admitted as acute medical emergencies between January 2002 and December 2014 (13-year peroid), were included for analysis, including those admitted into the intensive care or high-dependency units. The proportion of males was 48.5%. The median (IQR) length of stay (LOS) was calculated to be 5.9 (2.4, 13.0) days. The median (IQR) age was 64.5 (43.9, 78.5) years. A Charlson Comorbidity Score of 0, 1, or 2 was present in 45.9%, 27.4% and 26.7% respectively. Major disease categories (MDC) were respiratory (25.4%), neurological (16.8%), cardiovascular (16.1%), and gastrointestinal (10.2%). 

A respiratory patient was defined as a patient who had been referred to the pulmonary function laboratory for formal testing (20.7% of total episodes) or with a discharge diagnosis of chronic obstructive pulmonary disease (26.1%) or of pneumonia (6.7%); this gave a total of all episodes labelled as respiratory of 39.5%. 

The medical cohort, whose episode duration was <30 days, is tabulated by admission of non-respiratory or respiratory category (Table 1). The respiratory category were older: 68.4 years old (52.6, 78.6) vs. 56.4 years old (37.2, 75.8), had a longer length of stay 6.0 days (3.0, 10.9) vs. 4.4 days (1.7, 8.9) and a significantly worse outcome with 30-day in-hospital mortality 6.3% (95%CI: 6.0, 6.6) vs. 3.6% (95%CI: 3.4, 3.7). The respiratory category had a significantly higher occurrence of Acute Illness Severity, Charlson Co-Morbidity and Chronic Disabling Disease; however, there was a lower prevalence of sepsis.

### 3.2. Impact of Air Pollutants on In-Hospital Mortality (Figure 1, Figure 2, Figure 3)

**Figure 1:**
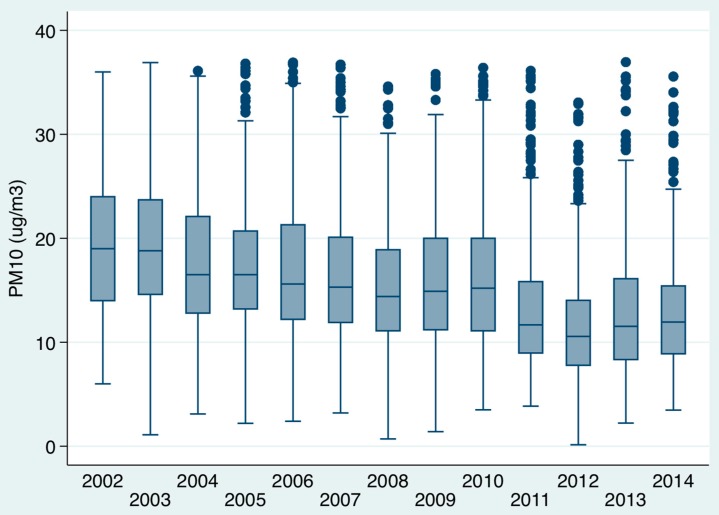
Trends of air pollution over time (PM10 µg/m^3^) from 2002 to 2014 represented as boxplots. Boxes indicate the interquartile ranges IQR; lines within boxes indicate medians; whiskers represent 5th and 95th percentile values, and circles represent outliers. The median and IQR are displayed for each year. The medians (IQR), for 2002–2003, were 19.0 (14.4, 23.7), for 2004–2007, were 16.1 (12.5, 21.1), and for 2008–2014, were 12.9 (9.3, 17.4), a significant fall over time.

**Figure 2:**
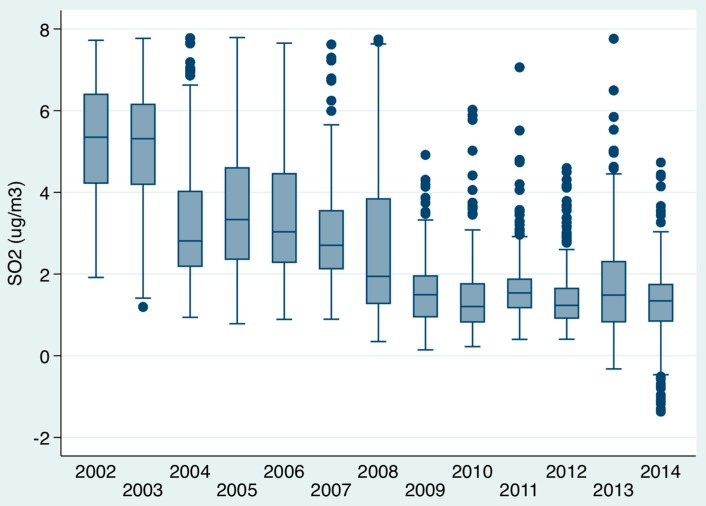
Trends of air pollution over time (SO_2_ µg/m^3^) from 2002 to 2014 represented as boxplots. Boxes indicate the IQR; lines within boxes indicate medians; whiskers represent 5th and 95th percentile values; and circles represent outliers. The medians (IQR) for 2002–2003 were 5.3 (4.3, 6.3), for 2004–2007, were 3.0 (2.3, 4.1), and for 2008–2014, were 1.4 (1.0, 2.0), a significant fall over time.

**Figure 3:**
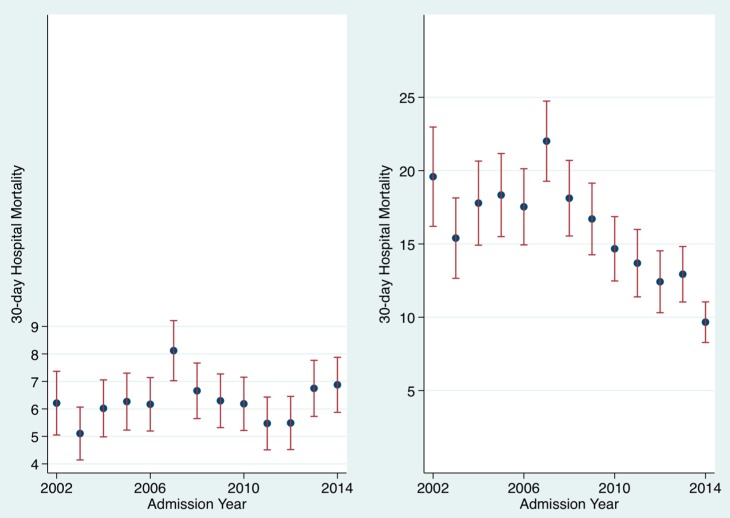
The in hospital mortality for respiratory admissions declined from 2002 to 2014. When calculated by episode in 2002 it was 6.2% (95% CI: 5.1, 7.4) and in 2014 6.9% (95% CI: 5.9, 7.9). However, calculated by unique patient (last admission if >1), the mortality figures were, for 2002, 19.6% (95% CI: 16.2%, 23.0%), and in 2014, 9.7% (95% CI: 8.3%, 11.0%).

There was a considerable reduction in air pollution levels over time between 2002 and 2014 (Figure 1 and Figure 2). The PM10 level (µg/m^3^) median and IQR levels for example in 2002–2003 was 19.0 (14.4, 23.7), by 2004–2007 these had fallen to 16.1 (12.5, 21.1) while 2008–2014 had medians of 12.9 (9.3, 17.4). The SO_2_ levels (µg/m^3^) also showed a significant fall over time; the medians (IQR) for 2002–2003 were 5.3 (4.3, 6.3), for 2004–2007 were 3.0 (2.3, 4.1) and 2008–2014 were 1.4 (1.0, 2.0). The mortality for respiratory admission between 2012 and 2014 fell considerably (Figure 3); considering unique patients only (last admission if > 1 episode), in 2002, respiratory admission mortality was 19.6% (95% CI: 16.2, 23.0) and, in 2014, 9.7% (95% CI: 8.3, 11.0). This fall was not evident for all respiratory episodes, in 2002 respiratory admission mortality by episode was 6.2% (95% CI: 5.0, 7.4) and in 2014 similar at 6.9% (95% CI: 5.9, 7.9). It is, however, important to remember that episodes count the same patients admitted repeatedly, the numerator (number of deaths), therefore, does not increase unlike the denominator (that is inflated–reducing the mortality estimate) in the calculation. Only 23.5% of respiratory patients were admitted once, 17.4% twice, 18.6% thrice, 10.8% four, and 7.1% five times; 29.9% were admitted at least five times, 9.8% at least 10 times, 2.4% at least twenty times, and 0.9% at least thirty times. For unique patients (last admission only counted if >1 admission) with a respiratory admission, between 2002 and 2014 the relative risk reduction of a 30-day in-hospital death was 50.7% with a numbers-to-treat value of 10.1

Patients were divided by quintile of the recorded air pollutant, particulate matter (PM10) and sulfur dioxide (SO_2_). The data represented the daily levels for the three measurement stations. For PM10 quintile, the median (IQR) increased from 8.1 μg/m^3^ (6.5, 9.2) to 32.0 μg/m^3^ (27.2, 40.8) from quintiles 1 to 5. Mortality by quintile was 14.0%, 13.5%, 14.9%, 17.4%, and 17.4%; mortality for quintiles 4 and 5 (compared with the base quintile) was significantly (*p* < 0.002) increased; univariate odds ratios (OR) of 1.30 (1.11–1.51) and 1.29 (1.10–1.51) were calculated, respectively.

Increasing mortality by quintile was also observed for SO_2_. The median and IQR increased from 0.83 (0.62, 1.00) μg/m^3^ to 6.40 (5.36, 9.03) μg/m^3^; mortalities by SO_2_ quintile were 12.8%, 13.9%, 16.3%, 17.0%, and 17.7%. Mortality for quintiles 3, 4, and 5 (the top three quintiles) appeared significantly (*p* < 0.001) increased as compared with the first quintile; univariate ORs of 1.32 (1.13–1.53), 1.39 (1.19–1.62) and 1.46 (1.24–1.71) were calculated, respectively.

### 3.3. Risk Estimates of Air Quality

The major predictors of a adverse outcome with a respiratory admission were acute illness severity [23,24,25], Charlson co-morbidity index [26], and chronic disabling disease [27], sepsis status [28], and deprivation index (SAHRU National Index) [29]. The model predicted any in-hospital death by day 30 with an area under the reciever operator curve (AUROC) of 0.83 (95% CI 0.82, 0.84). The prediction of the acute illness severity was OR 4.87 (95% CI: 4.11, 5.77), Charlson co-morbidity index 1.34 (95% CI: 1.22, 1.47), chronic disabling disease 1.29 (95% CI: 1.20, 1.38), sepsis 2.04 (95% CI: 1.84, 2.26), and deprivation index quintile 1.07 (95% CI: 1.01, 1.14). After adjustment for those aforementioned predictors, the PM10 level, measured on the patient admission day, was predictive of a 30-day in-hospital death for the 3rd to 5th PM10 quintiles, respectively, with adjusted ORs of 1.22 (95% CI: 1.00, 1.50), 1.33 (95% CI: 1.08, 1.63), and 1.45 (95% CI: 1.18, 1.78).

Similarly, SO_2_ was independently predictive of mortality for the 3rd, 4th, and 5th quintiles. SO_2_ levels measured on the patient admission day, even after predictor variable adjustment, remained predictive of 30-day in-hospital mortality for the 3rd, 4th and 5th quintiles, respectively, with adjusted ORs of 1.57 (95% CI:1.28, 1.92), 1.75 (95% CI:1.42, 2.15), and 1.91 (95% CI:1.53, 2.37).

### 3.4. Relationship between Underlying Level of Pulmonary Function and Outcome (Figure 4 and Figure 5)

Many of these patients had been referred to the pulmonary function laboratory with data available for 20.7% of all episodes (n = 17,095 admissions). Patients were divided into two groups according to their FEV_1_. We used a FEV_1_ cut off point of ≥2.0 L to examine the impact of the quintiles of PM10 and SO_2_ on the 30-day in-hospital mortality. There was a strong linear relationship between the mortality and the level of the pollutants on the day of the emergency medical admission. For those with better function, the mortality at the lowest PM10 quintile at time of admission was 6.5% (95% CI: 6.1, 6.9), but increased to 9.5% (95% CI: 9.0, 10.0) at the highest PM10 quintile. For the higher risk patients (FEV_1_ < 2.0 L), the mortality at the lowest PM10 quintile at time of admission was 9.9% (95% CI: 8.8, 10.9), but increased to 14.2% (95% CI: 12.8, 15.6) at the highest PM10 quintile.

Similarly for SO_2_ quintiles, there was a strong linear relationship between the mortality and the level of the pollutants on the day of the emergency medical admission. For those with better function, the mortality at the lowest SO_2_ quintile at time of admission was 5.9% (95% CI: 5.5, 6.2) but increased to 10.2% (95% CI: 9.7, 10.8) at the highest SO_2_ quintile. For the higher risk patients (FEV_1_ < 2.0 L), the mortality at the lowest SO_2_ quintile at time of admission was 9.2% (95% CI: 8.2, 10.2), but increased to 15.7% (95% CI: 14.1, 17.2) for the highest SO_2_ quintile.

## 4. Discussion

This study investigated the effect of low levels of PM10 and SO_2_ air pollutants on all-cause mortality for respiratory patients following an emergency medical admission, those admitted as in-patients having accessed the hospital through the emergency department. Much of the hospital specific literature has focused either on general acute admissions or on particular respiratory illnesses and cohorts [5,9,15,30,31]. Few, if any, studies have extensively examined respiratory patients in an entire emergency medical cohort and, as such, this work adds to the literature in this regard. For PM10 and SO_2_, respectively, it was evident that higher levels of air pollutants on the day of hospital admission, albeit relatively low as compared with levels in other European cities [2], predicted an adverse in-hospital outcome for respiratory patients. Given the range of patient acuity encountered for medical inpatients, it was necessary to account for illness severity through the use of a novel predictive multivariate model (AUROC = 0.83). Having adjusted for major outcome predictors, increased levels of PM10 and SO_2_ on the day of admission remained as predictors of 30-day in hospital mortality. Further, the relationship between a patient’s outcome and their underlying level of pulmonary function, as described using FEV_1_, showed those with lower pulmonary function scores to be at higher risk of in-hospital mortality when admitted on days of increased air pollutant levels. Thus, increased PM10 and SO_2_ air pollutant concentrations, even at low levels, increase the in-hospital mortality risk of respiratory patients with further elevated risks associated with poorer lung function.

Numerous studies have shown an association between concentrations of fine particulate matter and gas pollutants, such as PM10 and SO_2_ (µg/m^3^), and risk of death from all causes and from cardiovascular and respiratory illnesses [1,2,3,4,32] and indeed for acute cohorts [15]. The effect of such pollutants on human health may manifest as the result of long term exposure to poor air quality, or short term response to day-to-day variations in air quality [17], as was examined in this work. While studies has shown adverse outcomes to be evident even at low to medium levels [2,3,13,14,15], there remains a dearth of literature characterizing the effect of very low levels of air pollutants on human health, particularly those with respiratory illness. Studies on low levels of PM exist; however, their focus tends to be on city-wide analysis [32], with none providing the level of controllable and recordable parameters as is presented in this work for emergency medical admissions, The World Health Organization (WHO) has proposed air quality guidelines of 50 μg/m^3^ and 20 μg/m^3^ for 24-h averages and an annual mean PM10, respectively, and a 24-h average of 20 μg/m^3^ for SO_2_, while it is acknowledged that guidelines cannot completely protect against adverse health effects [16,17]. When considered in the context of this investigation, it is evident that average measurements within the catchment area were well below guidelines, suggesting the studied area to boast very good air quality. Indeed, since 2009, the 95th percentile SO_2_ concentrations were recorded to be at a level regarded as that of natural background concentrations for rural Europe, quoted as below 5μg/m^3^ [16]. Accordingly, while our data concurs with the literature, showing an increased air pollutant level to predict adverse outcome, the absolute levels of PM10 and SO_2_ detected were very low. The study adds to the literature in this regard [2,3,13,14,32] evidencing the continued impact of air quality on human health, even where air quality compares well with international guidance.

In addition to the effect of ambient air pollutants, forced expiratory volume in 1 second (FEV_1_) was shown to significantly predict in-hospital mortality for the examined respiratory cohort. FEV_1_, a measure of pulmonary function, has been shown to be inversely associated with mortality risk [33] and an important risk factor not only for cause-specific but overall mortality [33,34]. The presence of both obstructive and restrictive lung disease is regarded as a significant predictor of earlier death in the long term; however, the trend has also been identified over the short term [33,35]. The influence of between-day variations in air pollution on changes in FEV_1_ has shown a pattern of increased fine particulate concentrations affecting worsening lung function over time periods of less than a day [30]. In this study, it was not possible to investigate whether measured FEV_1_ levels were directly associated with air pollutant levels; however, the data shows that patients with reduced lung function admitted on days when pollutants were in the upper quintiles were at a significantly increased risk of mortality. To the author’s knowledge, this is the first study of its kind relating both the FEV_1_ and pollutant level with in-hospital mortality risk. It should be re-emphasized that the upper quintiles reported in this study fall within the international air quality guidelines [16], further begging the question as to whether current air quality guidelines protect those at most risk.

Of particular interest was that the respiratory cohort identified as older, with higher acuity, according to the acute illness severity score, and frequent readmissions. The mortality and length of stay of these patients was significantly increased when compared to those non-respiratory patients. The elderly cohort has been identified as more susceptible to air pollutants, including PM and SO_2_, attributable to the rise in chronic diseases affecting more elderly rather than younger people [36,37,38,39]. Further, the catchment area or our institution is one of high deprivation, with such areas shown to be more susceptible to increased respiratory illnesses and respiratory related admission rates [40]. Increased pollution exposures have associated with increased mortality and hospital admissions/readmissions, mainly due to exacerbations of chronic diseases or to respiratory tract infections [36,37,39]. One alarming statistic from this study is that just under 30% of the respiratory patients were admitted at least five times, while approximately 10% were admitted 10 times. This would have significant implications for hospitalization costs as a result of increased bed days and resource utilization following repeated readmissions; a further hospital cost investigation is merited. Having identified the association between air pollutants, respiratory illness, and mortality risk, it must be considered what interventions could be put in place to reduce adverse health effects. Although the studied air pollutants were shown to be independent predictors of admission and mortality, the absolute pollutants concentrations are regarded as low with the pollutant effect size considered to be small when compared with other more influential predictors. Consequently, in the context of this work, an interventional approach may benefit from a balance between air quality improvement and the encouragement of social mobility towards healthier living habits [40].

Interpreting this work with a global view may offer some value to institutions in developing countries which associated with a poorer air quality record [8,39,41] As discussed previously, after a governmental intervention in Dublin banning the marketing, sale and delivery of bituminous coal [12], a decrease in the black smoke concentration was affected, which led to a dramatic reduction in respiratory and cardiovascular death rates [12]. While the main source of pollution in Dublin was from coal use in homes, there was less influence from industrial sources of pollution. Accordingly, where an improvement in air quality was achievable, a similar affect may not be immediately possible in developing countries where there is an increased dependency on industry [8,39]. A balance between improving air quality, offering preventative measures and continued industrial productivity offers as a major challenge.

As with any study, it is important that limitations be noted and discussed. The limitations of this study included that there was a lack of infectious disease pandemics data which, as a result, were not corrected for. While the FEV_1_ data showed decreased lung function to affect mortality risk, it was not possible to directly associate air quality with FEV_1_. The recorded PM10 levels were reliant on two monitoring stations in the area with monitoring carried out only once per day for this pollutant. Further, given the length of the study period, therapy changes have not been accounted for. Our study included a large general “take”; however, it should be recognized that this is a single center study and, thus, its findings may not translate to other sites. Of importance, the correction for confounding factors that may affect mortality in medical admissions have been accounted for through the use of acute illness severity, co-morbidities, major disease category, and deprivation. 

## Figures and Tables

**Figure 4:**
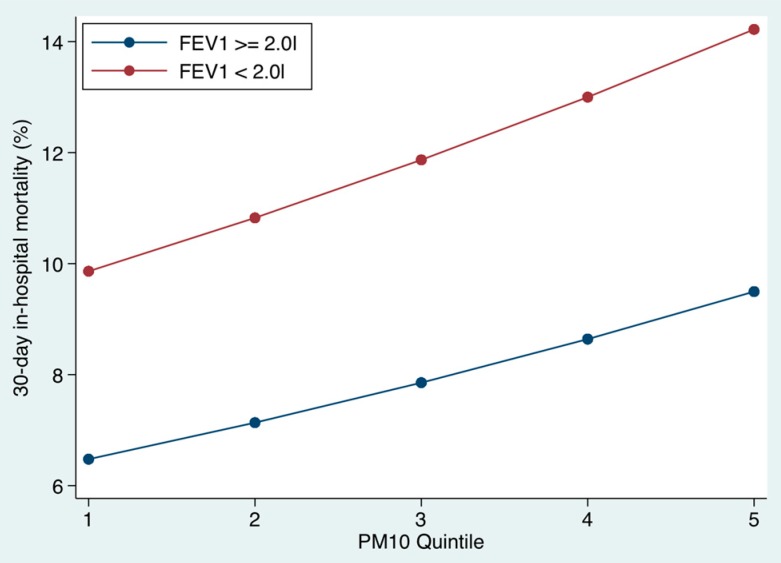
The 30-day in hospital mortality related to the underlying level of respiratory function (≥ or < 2.0 L forced expiratory volume (FEV_1_)). We divided PM10 levels by quintiles; the cut points were at 9.9, 13.1, 16.8, and 23.1 µg/m^3^, respectively. Mortality was clearly related to the underlying level of the PM10 on the day of hospital admission. The risk estimate was derived from the logistic regression multivariable model.

**Figure 5:**
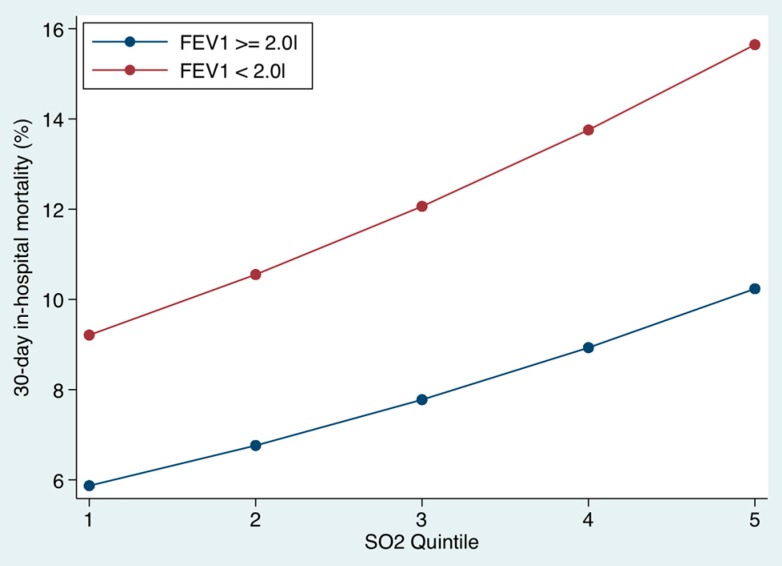
The 30-day in hospital mortality related to the underlying level of respiratory function (≥ or < 2.0 L FEV_1_). We divided sulfur dioxide (SO_2_) levels by quintiles; the cut points were at 1.1, 1.7, 2.5, and 4.3 µg/m^3^, respectively. Mortality was clearly related to the underlying level of the PM10 on the day of hospital admission. The risk estimate was derived from the logistic regression multivariable model.

**Table 1:** Characteristic of emergency admissions (2002–2014) by respiratory status.

Factor	Level	Non-Respiratory	Respiratory	*p*-Value
N		45,132	28,757	
Gender	Male	22,314 (49.4%)	13,757 (47.8%)	<0.001
	Female	22,818 (50.6%)	15,000 (52.2%)	
Outcome	Alive	43,527 (96.4%)	26,944 (93.7%)	<0.001
	Died	1605 (3.6%)	1813 (6.3%)	
Age, median interquartile ranges (IQR)		56.4 (37.2, 75.8)	68.4 (52.6, 78.6)	<0.001
Length of Stay (days)		4.4 (1.7, 8.9)	6.0 (3.0, 10.9)	<0.001
Admission Incidence		31.9 (19.6, 39.6)	32.6 (19.9, 40.1)	<0.001
Acute Illness Severity	1	1952 (4.8%)	281 (1.0%)	<0.001
	2	4188 (10.3%)	985 (3.7%)	
	3	6371 (15.7%)	2242 (8.3%)	
	4	7285 (18.0%)	4070 (15.1%)	
	5	7461 (18.4%)	5613 (20.9%)	
	6	13,278 (32.8%)	13,703 (51.0%)	
Charlson Index	0	26,630 (59.0%)	7302 (25.4%)	<0.001
	1	9100 (20.2%)	11,128 (38.7%)	
	2	9402 (20.8%)	10,327 (35.9%)	
Disabling Disease	0	7002 (15.5%)	1453 (5.1%)	<0.001
	1	12,712 (28.2%)	5693 (19.8%)	
	2	13,645 (30.2%)	7784 (27.1%)	
	3	7906 (17.5%)	7624 (26.5%)	
	4	3867 (8.6%)	6203 (21.6%)	
Sepsis Status	0	35,892 (79.5%)	20,046 (69.7%)	<0.001
	1	7816 (17.3%)	7552 (26.3%)	
	2	1424 (3.2%)	1159 (4.0%)	

## References

[1] Samet J.M., Dominici F., Curriero F.C., Coursac I., Zeger S.L. (2000). Fine particulate air pollution and mortality in 20 U.S. cities, 1987–1994. N. Engl. J. Med..

[2] Katsouyanni K., Touloumi G., Spix C., Schwartz J., Balducci F., Medina S., Rossi G., Wojtyniak B., Sunyer J., Bacharova L. (1997). Short-term effects of ambient sulphur dioxide and particulate matter on mortality in 12 European cities: Results from time series data from the APHEA project. Air Pollution and Health: A European Approach. Br. Med. J..

[3] Dockery D.W., Pope C.A. (1994). Acute respiratory effects of particulate air pollution. Annu. Rev. Public Health.

[4] Carugno M., Consonni D., Randi G., Catelan D., Grisotto L., Bertazzi P.A., Biggeri A., Baccini M. (2016). Air pollution exposure, cause-specific deaths and hospitalizations in a highly polluted Italian region. Environ Res..

[5] Pope C.A., Kanner R.E. (1993). Acute Effects of PM10 Pollution on Pulmonary Function of Smokers with Mild to Moderate Chronic Obstructive Pulmonary Disease. Am. Rev. Respir. Dis..

[6] Atkinson R.W., Anderson H.R., Sunyer J., Ayres J., Baccini M., Vonk J.M., Boumghar A., Forastiere F., Forsberg B., Touloumi G. (2001). Acute effects of particulate air pollution on respiratory admissions: Results from APHEA 2 project. Air Pollution and Health: A European Approach. Am. J. Respir. Crit. Care Med..

[7] Dominici F., Peng R.D., Bell M.L., Pham L., McDermott A., Zeger S.L., Samet J.M. (2006). Fine particulate air pollution and hospital admission for cardiovascular and respiratory diseases. J. Am. Med. Assoc..

[8] Phung D., Hien T.T., Linh H.N., Luong L.M., Morawska L., Chu C., Binh N.D., Thai P.K. (2016). Air pollution and risk of respiratory and cardiovascular hospitalizations in the most populous city in Vietnam. Sci. Total Environ..

[9] Sunyer J., Schwartz J., Tobias A., Macfarlane D., Garcia J., Anto J.M. (2000). Patients with chronic obstructive pulmonary disease are at increased risk of death associated with urban particle air pollution: A case-crossover analysis. Am. J. Epidemiol..

[10] Schwartz J., Dockery D.W. (1992). Increased mortality in Philadelphia associated with daily air pollution concentrations. Am. Rev. Respire. Dis..

[11] Kelly I., Clancy L. (1984). Mortality in a general hospital and urban air pollution. Ir. Med. J..

[12] Clancy L., Goodman P., Sinclair H., Dockery D.W. (2002). Effect of air-pollution control on death rates in Dublin, Ireland: An intervention study. Lancet.

[13] Schwartz J. (1994). Air pollution and daily mortality: A review and meta analysis. Environ. Res..

[14] Katsouyanni K., Zmirou D., Spix C., Sunyer J., Schouten J.P., Ponka A., Medina S., Bachárová L., Anderson H.R. (1995). Short-term effects of air pollution on health: A European approach using epidemiological time-series data. The APHEA protocol. Eur. Respir. J..

[15] Lyons J., Chotirmall S.H., O’Riordan D., Silke B. (2014). Air quality impacts mortality in acute medical admissions. Q. J. Med..

[16] World Health Organisation (WHO) (2005). Air Quality Guidelines, Global Update 2005, Particulate Matter, Ozone, Nitrogen Dioxide and Sulphur Dioxide.

[17] Brunekreef B., Holgate S.T. (2002). Air pollution and health. Lancet.

[18] Rooney T., Bennett K., Silke B. (2008). Reduced mortality and length of stay in a teaching hospital after establishment of an acute medical admission unit (AMAU): A 5-year prospective study. Eur. J. Int. Med..

[19] Rooney T., Moloney E.D., Bennett K., O’Riordan D., Silke B. (2008). Impact of an acute medical admission unit on hospital mortality: A 5-year prospective study. Q. J. Med..

[20] Conway R., O’Riordan D., Silke B. (2013). Long-term outcome of an AMAU—A decade’s experience. Q. J. Med..

[21] O’Loughlin R., Allwright S., Barry J., Kelly A., Teljeur C. (2005). Using HIPE data as a research and planning tool. Ir. J. Med. Sci..

[22] Keary J., Jennings S.G., O’Connor T.C., McManus B., Lee M. (1998). PM10 concentration measurements in Dublin city. Environ. Monit. Assess..

[23] Froom P., Shimoni Z. (2006). Prediction of hospital mortality rates by admission laboratory tests. Clin. Chem..

[24] O’Sullivan E., Callely E., O’Riordan D., Bennett K., Silke B. (2012). Predicting outcomes in emergency medical admissions—Role of laboratory data and co-morbidity. Acute Med..

[25] Prytherch D.R., Sirl J.S., Schmidt P., Featherstone P.I., Weaver P.C., Smith G.B. (2005). The use of routine laboratory data to predict in-hospital death in medical admissions. Resuscitation.

[26] Charlson M.E., Pompei P., Ales K.L., MacKenzie C.R. (1987). A new method of classifying prognostic comorbidity in longitudinal studies: Development and validation. J. Chronic Dis..

[27] Chotirmall S.H., Picardo S., Lyons J., D’Alton M., O’Riordan D., Silke B. (2014). Disabling disease codes predict worse outcomes for acute medical admissions. Int. Med. J..

[28] Chotirmall S.H., Callaly E., Lyons J., O’Connell B., Kelleher M., Byrne D., O’Riordan D., Silke B. (2014). Blood cultures in emergency medical admissions: A key patient cohort. Eur. J. Emerg. Med..

[29] Kelly A., Teljeur C. (2007). The National Deprivation Index for Health Services Research.

[30] Dales R., Chen L., Frescura A.M., Liu L., Villeneuve P.J. (2009). Acute effects of outdoor air pollution on forced expiratory volume in 1 s: A panel study of schoolchildren with asthma. Eur. Respir. J..

[31] Peacock J.L., Anderson H.R., Bremner S.A., Marston L., Seemungal T.A., Strachan D.P., Wedzicha J.A. (2011). Outdoor air pollution and respiratory health in patients with COPD. Thorax.

[32] Broome R.A., Fann N., Cristina T.J., Fulcher C., Duc H., Morgan G.G. (2015). The health benefits of reducing air pollution in Sydney, Australia. Environ. Res..

[33] Gruer L., Hart C.L., Watt G.C. (2015). After 50 years and 200 papers, what can the Midspan cohort studies tell us about our mortality?. Public Health.

[34] Hebert J.R., Pednekar M.S., Gupta P.C. (2010). Forced expiratory volume predicts all-cause and cancer mortality in Mumbai, India: Results from a population-based cohort study. Int. J. Epidemiol..

[35] Mannino D.M., Buist A.S., Petty T.L., Enright P.L., Redd S.C. (2003). Lung function and mortality in the United States: Data from the First National Health and Nutrition Examination Survey follow up study. Thorax.

[36] Simoni M., Baldacci S., Maio S., Cerrai S., Sarno G., Viegi G. (2015). Adverse effects of outdoor pollution in the elderly. J. Thorac. Dis..

[37] Fung K.Y., Khan S., Krewski D., Chen Y. (2006). Association between air pollution and multiple respiratory hospitalizations among the elderly in Vancouver, Canada. Inhal. Toxicol..

[38] Namdeo A., Tiwary A., Farrow E. (2011). Estimation of age-related vulnerability to air pollution: Assessment of respiratory health at local scale. Environ. Int..

[39] Xu Q., Li X., Wang S., Wang C., Huang F., Gao Q., Wu L., Tao L., Guo J., Wang W. (2016). Fine Particulate Air Pollution and Hospital Emergency Room Visits for Respiratory Disease in Urban Areas in Beijing, China, in 2013. PLoS ONE.

[40] Hawker J.I., Olowokure B., Sufi F., Weinberg J., Gill N., Wilson R.C. (2003). Social deprivation and hospital admission for respiratory infection: An ecological study. Respir. Med..

[41] Qiu H., Tian L.W., Pun V.C., Ho K.F., Wong T.W., Yu I.T. (2014). Coarse particulate matter associated with increased risk of emergency hospital admissions for pneumonia in Hong Kong. Thorax.

